# Challenges of E-Waste Dismantling in China

**DOI:** 10.3390/toxics12120867

**Published:** 2024-11-28

**Authors:** Bitong Li, Dongling Liu, Lina Zhang, Yue Wu, Xianlin Ding, Xiang Zeng

**Affiliations:** 1 School of Public Health, Zhejiang Chinese Medical University, 548 Binwen Road, Hangzhou 310053, China; 202311116911009@zcmu.edu.cn (B.L.); zhanglina@zcmu.edu.cn (L.Z.); wuyue@zcmu.edu.cn (Y.W.); 2 School of Basic Medical Science, Zhejiang Chinese Medical University, 548 Binwen Road, Hangzhou 310053, China; liudongling@zcmu.edu.cn; 3 Luqiao District Center for Disease Control and Prevention, 208 Lingshan Road, Taizhou 318050, China; 4 Zhejiang International Science and Technology Cooperation Base of Air Pollution and Health, 548 Binwen Road, Hangzhou 310053, China

**Keywords:** e-waste, recycling, dismantling, circular parks, internet plus, China

## Abstract

Electronic and electrical products have deeply permeated all aspects of life, bringing a lot of convenience to individuals. However, the generation of e-waste after their end-of-life has resulted in serious risks both to the ecological environment and human health due to a lack of scientific and effective recycling and treatments. As two major types of components in e-waste, heavy metals and plastics can not only directly enter the human body via inhalation, ingestion, and skin absorption, but also accumulate in the human body indirectly through the food chain. E-waste is full of resources such as valuable metals like gold, silver, and copper that are often discarded incorrectly. Environmental and health risks derived from unregulated e-waste dismantling activities may be finally addressed through the application of advanced e-waste recycling technology, policy support of governments, legislation on recycling laws and regulations, and the improvement of public environmental protection awareness. This review gives a brief overview of the history, current situation, and future development of e-waste in China, which may provide novel thinking and approaches to e-waste management in the world.

## 1. Introduction

E-waste, well known as waste from electronic and electrical equipment (WEEE), refers to discarded or end-of-life electronic products and electrical appliances [1]. Global e-waste is one of the fastest-growing waste streams, which arouses sustained public concern and debate [2]. It is estimated that the global e-waste generated was 62 million tons in 2022 (7.8 kg per capita) and 82 million tons in 2030 (9.0 kg per capita) [3]. This rapid growth of e-waste can be primarily attributed to a leap forward in the electronics industry, the upgrading of consumer preferences, and informal e-waste recycling activities. Although e-waste contains a variety of valuable raw materials like gold, silver, and copper, that were estimated to be worth approximately 60 billion dollars [3], only about 22.3% of global e-waste is collected and properly recycled each year [3]. In other words, most e-waste either ends up in open dumpsites, is illegally buried, or is informally recycled in real life. Therefore, it is necessary to appeal to society and the government to improve the e-waste recycling rate, to develop advanced e-waste recycling technology, to reduce e-waste emissions, to raise e-waste management efficiency, and to cultivate environmental awareness.

**Figure 1:**
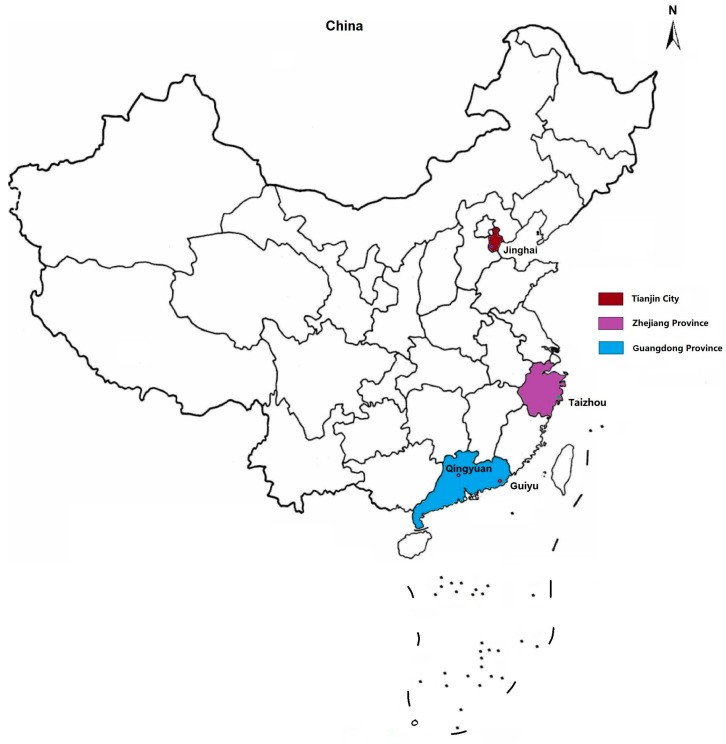
Major locations of world-class e-waste recycling centers in China.

Different from general solid waste, e-waste is mainly comprised of metals (60%) and plastics (30%) some of which are well-known for their high toxicity [4]; for example, approximately 2.7% of the metal ions belong to toxic metals [5]. Currently, the common informal e-waste dismantling and recycling activities include open-air burning, acid bathing, roasting, dismantling, shredding, dumping, and the disposal of non-resalable goods [6,7,8]. These types of e-waste treatment can release a large number of pollutants to the local environment, resulting in direct human exposure and indirect enrichment through the food chain. There are multiple world-class e-waste dismantling centers, such as Guiyu, Taizhou, Jinghai in China [9] (Figure 1). Notably, China is not only the largest electronic industrial country, but also the leading e-waste producer in the world with an approximated 20% annual growth rate [10]. Although China generates far more e-waste than other countries or regions in total (12 million metric tons/year), the per capita amount of e-waste is still well below the global average level (7.8 kg per capita) and almost one-third of that of Europe (17.6 kg per capita) [3]. This may indicate that China will bear a greater e-waste burden while facing a continuously growing e-waste challenge.

How to manage e-waste and, at the same time, protect the environment and public health has become an urgent problem to be solved in the present era of e-waste being rampant. As the former largest importer of e-waste and the current largest producer of e-waste in the world, China has been in a dilemma for a period of time when confronting domestic and overseas e-waste. In recent years, China has made significant progress in e-waste management [11,12], which may provide some reference for other countries and regions. However, there are still many obstacles to overcome in future. The objective of the review is to provide insights into the current state of e-waste recycling in China, with the intention of offering a reference or an experience for other countries worldwide in managing e-waste. This endeavor aims to ensure that the useful resources contained within e-waste can be more effectively utilized for the benefit of humanity. This review briefly summarizes the efforts and challenges relating to e-waste in China.

## 2. Compositions of E-Waste

**Figure 2:**
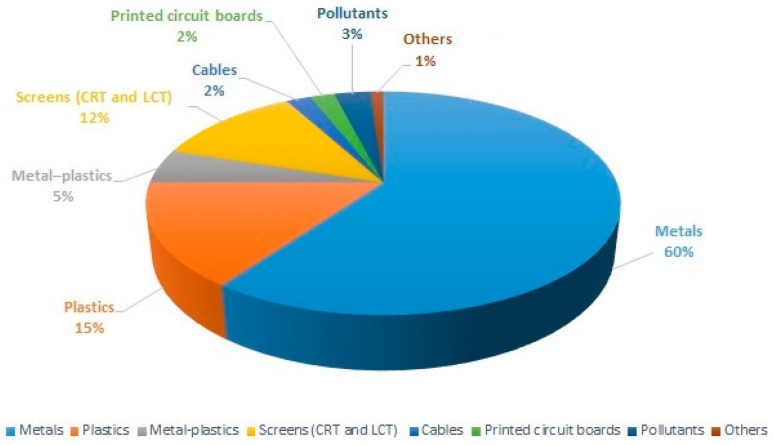
Composition and proportion of e-waste (adapted from [5]).

E-waste primarily comprises of metals, plastics, screens, metal–plastics, printed circuit boards, and cables. The proportion of each component varies (Figure 2). The toxic substances are mainly metal, organic materials, and screens. Printed circuit boards (PCB) are the main components of electronic products, and their components are mainly 305 kinds of plastics, 30% refractory oxides and enols, and 40% metals (of which copper accounts for 10–30%) [13]. As a result, WEEE has a complex, diverse, and heterogeneous composition, including steel (50%), non-ferrous metals (13%), plastics (21%), and other components (16%) such as ceramics, glass, and rubber [14]. Copper, zinc, and nickel are non-ferrous metals and are abundant in WEEE [15]. In addition, the plastic fraction of e-waste is further categorized into Acrylonitrile Butadiene Styrene (ABS), High Impact Polystyrene (HIP), Polycarbonate (PC), and Polypropylene (PP), which can be recycled separately [16]. Moreover, e-waste covers a wide range of electronic equipment and products. Of these, electronic equipment can be divided into six different categories, ranging from large household appliances, information technology and telecommunication equipment, lighting equipment, medical equipment, surveillance instruments, to vending machines. As for consumer electronic products, this category is composed of appliances, electronic tools, toys, leisure and sports equipment, as well as mobile phones and computers [17]. It should be noted that the electrical and electronic components of batteries, cables, printed circuit boards, plastic casings, cathode ray tubes, activated glass, and lead capacitors are also classified as e-waste upgrading based on previous European Union classification [18,19].

## 3. Generation and Cost of E-Waste

**Figure 3:**
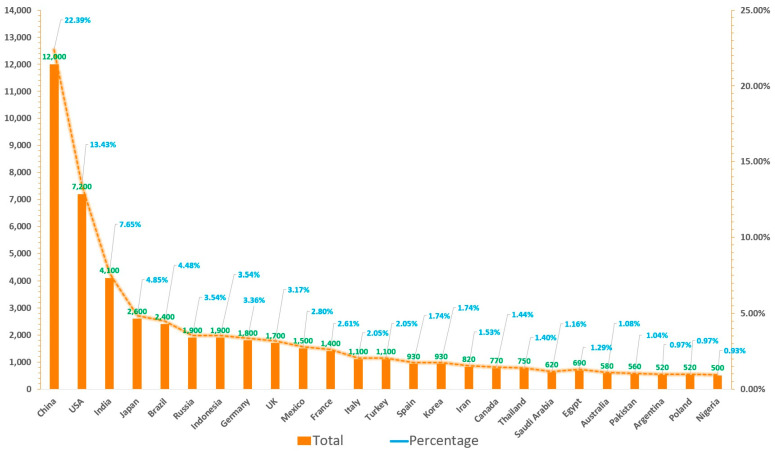
The total and percentage of e-waste generated from top 25 countries globally in 2024 (Data originally derived from [3]).

**Figure 4:**
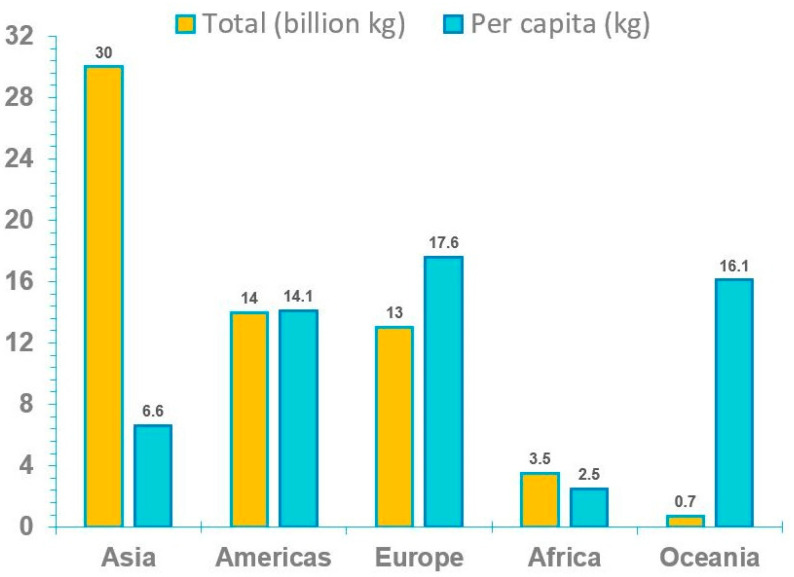
Total amount and the amount per capita of e-waste generated among the five continents of Asia, the Americas, Europe, Africa, and Oceania in 2024 (Data originally derived from [3]). The order by total amount of e-waste was Asia (24.9 Mt) > Americas (13.1 Mt) > Europe (12.0 Mt) > Africa (2.9 Mt) > Oceania (0.7 Mt); while the amount per capita of e-waste was Europe (16.2 kg per capita) > Oceania (16.1 kg per capita) > Americas (13.3 kg per capita) > Asia (5.6 kg per capita) > Africa (2.5 kg per capita).

**Figure 5:**
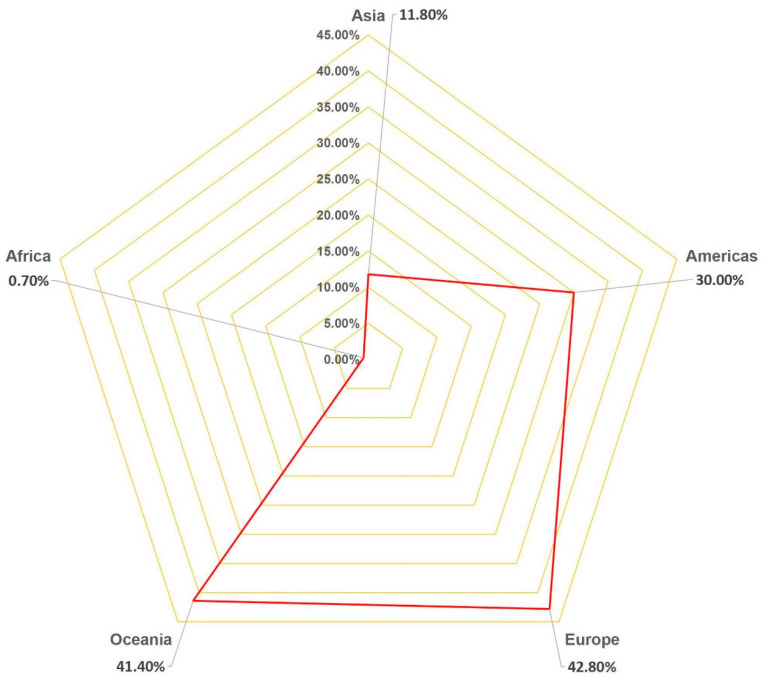
E-waste collection and recycling rates among the five continents of Asia, the Americas, Europe, Africa, and Oceania in 2022 using radar chart (Data originally derived from [3]). The order is Europe (42.8%) > Oceania (41.4%) > Americas (30.0%) > Asia (11.8%) > Africa (0.70%).

**Figure 6:**
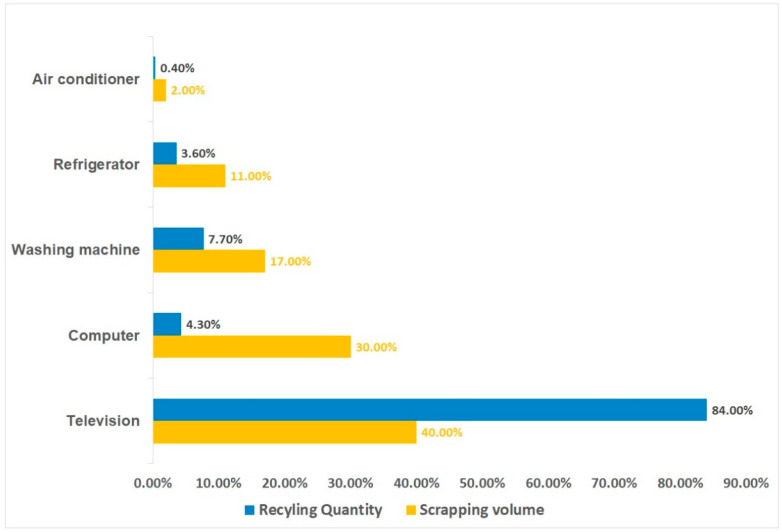
The proportion of theoretic recycling and scrapping for household e-waste in China (Data originally derived from [20]).

Estimating the quantity of e-waste generated is a leading and crucial step for e-waste management which is beneficial for understanding global e-waste flow trajectory. According to a United Nations University report titled “The Global E-waste Monitor Report 2024”, the amount of global e-waste climbed to a record of 62 million tons in 2022, which accounts for approximately 5% of the total solid waste produced worldwide [3]. While developed nations such as the United States and most European countries are still considered to be the main manufacturers and customers of e-waste globally, the scenario has changed, and some major developing countries such as China, India, Brazil, Russia, and Indonesia, have become the first, third, fifth, sixth, and seventh largest producers of e-waste (Figure 3). Based on the total and average volumes of e-waste for the five continents, it is evident that Asia has the highest total production of e-waste, while its per capita production is still at a very low level. On the other hand, Oceania has a low total production but a high per capita production (Figure 4). As for the collection recovery rates of these continents, Europe has the highest e-waste recycling rate among the five continents, surpassing the levels of other continents significantly, while Asia has the second highest e-waste recycling rate in the world (Figure 5). This next section illustrates the generation, growth, and recycling of various categories of e-waste. Notably, small household appliances contribute to the largest volume of e-waste, while heat exchangers exhibit the highest growth rate (Figure S1). Furthermore, televisions stand out for having both the highest percentage of end-of-life and recycling rates among household appliances. Conversely, air conditioners display the opposite scenario, with a low percentage of end-of-life and recycling rates (Figure 6) [20]. A comprehensive cost and revenue analysis of an e-waste recycling facility in California reveals that the primary cost driver for operations is material costs, which can constitute up to 37% of total expenses. This includes the outsourcing cost for cathode-ray tube (CRT) recycling, estimated at $0.33 per kg. The second most significant cost driver is labor costs, accounting for as much as 28% of total expenditures when management fees are excluded from consideration. Additional factors influencing costs include transportation, construction, and equipment expenses [21]. In terms of unit operation sorting by cost price, CRT glass recycling ranks highest, followed by classification, collection, and disassembly. From a revenue standpoint, the largest source of income derives from fees charged to customers; the second major source comes from metal recycling activities [21].

## 4. Flow of E-Waste

A necessary step for e-waste management is to estimate its flow, both domestically and cross-border. It seems that the main trend in e-waste transportation is from developed countries to developing countries [3]. About 5.1 billion kg of e-waste flows across national borders each year. Of this, 3.3 billion kg (65%) is an uncontrolled cross-border movement from high-income countries to low- and middle-income countries. Uncontrolled shipments may contain from 33 to 70 percent e-waste, with most controlled transboundary movements occurring within or to Europe and East Asia [3]. The remaining 1.86 billion kg (35% of total transboundary movements) is transported in the form of controlled movements. The bulk of e-waste comes mainly from western Europe (34.8 million kg), North America (29 million kg), Northern Europe (11.6 million kg), and Southeast Asia (9.9 million kg) [11]. One of the most critical reasons for the transboundary movement of e-waste is that the cost of metal treatment/extraction exceeds the potential value generated by treatment/extraction in the country of origin [21]. Additionally, these countries have stringent environmental protection laws and regulations concerning the disposal of waste or unprocessed materials from the recycling process, making the process very costly [22]. Moreover, the overall cross-border flows are economically beneficial to the developing countries because of their lower labor costs and poorly regulated dispositions [21]. From the perspective of collection, semi-formal and informal extraction of heavy metals from e-waste is common in developing countries, and recycled heavy metals are primarily sold to the formal industry for overseas sale [23]. However, this informal recycling of e-waste finds its way to developed countries with the establishment of highly capitalized, state-of-the-art operations for processing various fractions and components of e-waste in developed countries. Consequently, there are substantial cross-border waste movements [16]. The main source countries include the USA, Canada, Australia, the UK, Germany, France, the Netherlands, and Belgium. The main destination countries consist of China, India, Malaysia, Indonesia, the Philippines, Ghana, Nigeria, and Mexico (Figure S2) [24,25,26,27]. However, the data on the flow of e-waste within individual countries is very limited and remains unclear.

## 5. Environmental Impact of E-Waste in China

E-waste is known to contain a range of toxic substances, such as metals and plastics. Informal e-waste recycling methods such as burning, roasting, acid leaching, shredding, and dumping contribute to the release of these toxins into various environmental media, such as soil, air, water, dust, and sediment [28]. These environmental media are crucial for the local residents’ survival (Table 1). Additionally, the movement and transformation of pollutants from e-waste across different environmental media and regions raise concerns as they can impact other regions and populations, particularly due to improper recycling practices and illegal disposal operations [9]. Furthermore, occupational exposure occurs when informal recycling workers engage in e-waste recycling without appropriate protective gear [29].

### 5.1. Soil

In recent decades, as human activities have increased, hazardous substances have also been released substantially into the soil, especially agricultural soil that is heavily polluted [9]. The route via which humans are exposed to heavy metals within any given farming system are primarily determined by food habits [30]. Given that hazardous compounds, particularly heavy metals, can be absorbed by plants through their roots and then transferred to the stem and leaf, soil is thought to be a major source of these pollutants in crops and vegetables [31]. The metal concentrations near an abandoned e-waste recycling site was estimated in a previous study, which found varying levels of contamination in different areas. In particular, heavy metal concentrations were higher in the samples collected from the burnt site than those from the paddy field and stream, especially cadmium (Cd), copper (Cu), zinc (Zn), lead (Pb), nickel (Ni), barium (Ba), stibium (Sb), stannum (Sn), and argentum (Ag) [32,33]. A study conducted in the Ziya e-waste dismantling area in Tianjin, China found notable concentrations of heavy metals, including Cu, Pb, chromium (Cr), Zn, Ni, and mercury in the soil, indicating significant heavy metal pollution [34]. In addition, soils from this area are known to release polychlorinated biphenyls (PCBs), polycyclic aromatic hydrocarbons (PAHs), polychlorinated dibenzo-p-dioxins and dibenzofurans (PCDD/Fs) into the environment [35]. These pollutants pose various hazards to the environment and human health, since PCDD/Fs are known as lipophilic compounds that can be bioaccumulated in the human body through the food web, while PCBs are frequently utilized as plasticizers, lubricants, and coolants in transformers and condensers, which contribute to environmental issues such as acid rain and ozone layer depletion [36]. Moreover, open burning and acid washing have significant impacts on soil microorganisms [37]. The levels of polybrominated biphenyl (PBB, 27.18 ng/g) were found to be substantially higher in soil samples taken from e-waste disassembly facilities in Zhejiang Province, China, compared to the control site [38]. Furthermore, per- and polyfluoroalkyl substance (PFAS) concentrations in Guiyu soil were reported to range between 0.44 and 141.27 ng/g, with concentrations of perfluoroalkyl sulfonic acid (PFOS) as high as 141.27 ng/g [39]. The presence of heavy metals in contaminated soil affects the structure and diversity of microbial communities, leading to significant variations in bacterial taxonomic composition between contaminated and uncontaminated areas [40]. By collecting e-waste contaminated soil samples from China and Pakistan and analyzing indigenous microbial communities, the results revealed that the microbial community composition and diversity, at both whole and core community levels, were affected significantly by PAHs, polybrominated diphenyl ethers (PBDEs), and heavy metals [41].

### 5.2. Air

Similarly, in terms of air pollution, e-waste pollutants are released into the atmosphere as dust or fumes, creating risks to human health through various exposure routes such as skin absorption and respiratory inhalation [42]. Studies have confirmed severe particulate matter (PM) and persistent organic pollutants (POPs) in China’s e-waste recycling zones since the 2010s. Operations in Guiyu, such as circuit board baking and plastics recycling, have resulted in low air quality and elevated polychlorinated dibenzo-p-dioxins and dibenzofurans (PCDD/Fs) levels (0.73~2.43 pg I-TEQ/m^3^). Other pollutants, such as Zn, Cr, PAHs, and polybrominated dibenzo-p-dioxins and dibenzofurans (PBDD/Fs), have also been found to be significantly higher in Guiyu. For example, atmospheric levels of Cu, Zn, and Cr in Guiyu were 483, 1038, and 1161 ng/m^3^, respectively, which were 4–33 times higher than those in other Asian nations [43]. In addition, the concentrations of atmospheric PCDD/Fs and PBDD/Fs in Guiyu were 64.9–2365 pg/m^3^ and 8.12–461 pg/m^3^, respectively, which were 12–18 times higher than those in the adjacent town of Chendian, and 37–133 times higher than those in nearby Guangzhou [44]. The range of atmospheric phthalates esters (PAE) concentrations at e-waste recycling sites was 200–1200 ng/m^3^, which was approximately 1–2 times greater than surrounding metropolitan regions [45]. A study conducted in Guiyu has shown that the average value of PM_2.5_ was 62.1 mg/m^3^, and the average levels of Pb, Cd, Cr, and manganese (Mn) in PM_2.5_ were 444 ng/m^3^, 7.25 ng/m^3^, 1161 ng/m^3^, and 60.6 ng/m^3^, respectively. These levels were found to be higher than those observed in most other regions of Asia [43]. One of our previous studies reported that the average PM_2.5_ concentration and Pb and Cd concentrations in PM_2.5_ in Guiyu were higher than that in the reference area, whereas there was no significant difference in Cr and Mn levels between the two areas [46]. Additionally, higher blood Pb and Cd levels were found in the children of Guiyu compared to their peers of the reference area. Our recent research results demonstrate that total atmospheric concentrations of PFASs in Guiyu ranged from 0.39 to 90.26 ng/g, and part of PFAS homologs were significantly higher in Guiyu than in the reference group [39].

### 5.3. Water

Contaminants from e-waste can also enter aquatic systems, such as groundwater and surface water, through leaching and diffusion processes, affecting adjacent aquatic organisms and potentially contaminating drinking water sources [9]. Informal disposal of e-waste can also result in acid emissions into soil and water bodies, while open burning releases pollutants that ultimately contaminate aquatic systems [47]. Wang et al. found that Pb concentration in the water from Guiyu reached 0.4 mg/L, which exceeded 8 times the limit [48]. Wu et al. observed that Cd and Mn levels in the wells in Longtang, south China exceeded the guideline values. Pond water was found to be severely acidified and contaminated by heavy metals, indicating the impacts of previous e-waste recycling activity [34]. Further research confirmed that the concentrations of metals in the groundwater were significantly higher in the e-waste areas compared to non-e-waste areas in south China [49]. The groundwater in the Qingyuan e-waste dismantling area is also contaminated, which deserves attention because some of the local residents still use groundwater as drinking water [29]. One study found that the levels of hazardous metals in the Lianjiang and Nanya Rivers were significantly higher than those detected at the surface in the underground streams of Guiyu, in contrast to Guiyu River [50]. In the Lianjiang river, running through Guiyu, PFAS were detected, which was found to be significantly more contaminated in the water in the Guiyu e-waste recycling area than in Haojiang [39]. Zhang et al.’s study found higher concentrations of heavy metals in crops near pools and in human urine samples near e-waste recycling stations [29]. Polybrominated diphenyl ethers (PBDEs), pentabromobenzene (PBB), and pentabromotoluene (PBT) were detected in all the water and sediment samples in Taizhou, indicating ubiquitous contamination in the aquatic environment in Taizhou [51]. Overall, high levels of heavy metals and contaminants in groundwater, surface water, crops, and even human urine samples were documented based on e-waste recycling areas in China. These findings highlight the urgent need for proper waste management practices and regulations to mitigate and prevent further environmental and health risks associated with e-waste contamination.

Unlike the aforementioned impacts, e-waste recycling not only recovers valuable metal resources but also generates additional employment opportunities. By recycling electronic waste to generate secondary raw materials, we have conserved 900 billion kg of minerals that would otherwise have been extracted through primary mining processes. This initiative has also led to the avoidance of 5.2 billion kg of carbon dioxide equivalent emissions, mitigating climate change and its impacts [3,52,53].

## 6. Health Effect of E-Waste

It is believed that individuals who live close to e-waste removal facilities are frequently and habitually exposed to the hazardous materials that are released and travel through a variety of natural routes [54]. E-waste discharges harmful toxic compounds that can be accumulated in plants and animal bodies and eventually in the long run find their way into the human body through the food chain. This undoubtedly has a negative impact on the health of nearby individuals [55]. For example, mercury tends to accumulate in food chain organisms to be converted to inorganic mercury of methylmercury, and methylmercury is more prone to being accumulated through food chains. Compared to non-coastal residents, coastal residents generally consume more freshwater products and seafoods, such as fish and shellfish, which means that coastal residents may face a higher risk of dietary exposure to mercury because even low-dose mercury exposure can have adverse effects on the nervous system in the human body [56]. Cr, PBDEs, and dioxins can result in not only noncancerous consequences such diabetes, hypertension, and atherosclerosis, but also cancer. Notably, the exposure of occupational workers in the e-waste dismantling area cannot be ignored. A study conducted in Guiyu has discovered that e-waste recycling workers were considerably more exposed to harmful toxins compared to the general population [47,57]. The study also found similar results when comparing atmospheric levels of PBDEs, indicating that the severe air pollution could have a direct impact on humans. Furthermore, it is crucial to acknowledge that the effects of e-waste contamination are not limited to occupational workers alone. Children and pregnant women residing in these areas are equally affected by hazardous exposure [57].

Due to their smaller size and higher ingestion rate (200 mg/kg/day) employed in risk assessments, children at all dismantling locations are presumably at greater health risk than adults [58]. Children’s lung function deteriorates with prolonged exposure to contaminated air, especially those who are going through a fast developmental period [28]. In terms of developmental toxicity, lead prevents calcium, iron, and zinc from being absorbed, which impairs children’s physical growth and development by inhibiting the manufacture and usage of hormones such as thyroid-stimulating hormone (TSH) and insulin-like growth factor (IGF). In Guiyu, physical characteristics like height, weight, and body mass index were negatively associated with children’s blood lead levels [59] and physical parameters such as height, weight, and body mass index [4]. Additionally, in local youngsters, exposure to Mn or Ni was associated with lower levels of the above-mentioned physical growth and development indicators [60]. It is hard for pregnant women residing in the regions where e-waste recycling occurs to protect themselves from heavy metals and organic pollutants found in e-waste. Thus, there is a more noteworthy possibility of premature births, smaller babies and lower birth weights, spontaneous abortions, stillbirths, lower Apgar scores, and lower neonatal behavioral neurological assessment (NBNA) scores [10,61,62].

## 7. E-Waste Management Policies and Regulations in China

The rapid growth of e-waste has become an environmental and human health challenge globally, especially in developing countries such as China, India, and Brazil. It is essential to strictly implement rules and regulations at ground level to effectively manage e-waste. The comprehensive implementation of laws and regulations is necessary for the efficient management of e-waste [63]. In China, a number of laws connected to the management of e-waste recycling and pollution prevention have been developed during the past twenty years (Table 2) and we have listed other important regulations from around the world. Strict legal frameworks for e-waste management, founded on the Extended Producer Responsibility (EPR) concept, are essential to the long-term growth of China’s e-waste recycling industry. The producer should be responsible for the entire life cycle of the product, especially in the recovery, treatment, and recycling stages after the product is discarded, following the principle of “whoever produces, whoever is responsible” [11]. In addition, the 2004-enacted Measures for the Administration of Permit for Operation of Hazardous Wastes are constantly being revised and adjusted to improve oversight and control over the gathering, storing, and disposal of hazardous waste, including abandoned circuit boards. Furthermore, the Chinese government launched the “Household Appliances Old for New Rebate Program” (Old for New Program) in June 2009, initially in four provinces and five major cities before its expansion nationwide. This program governs China’s electrical and electronic equipment recycling and disposal. The Management Regulation on the Recycling of Waste Electrical and Electronic Products (WEEE regulation) went into effect on 1 January 2011, supported by technical guidelines and directives [64]. With the implementation of the WEEE Treatment Fund Policy in July 2012, China’s e-waste treatment system received a significant boost. With the introduction of the “Old for New Program” and particularly the “Fund Policy”, the formal e-waste treatment system has been significantly advanced [65]. The regulation has established a catalogue system for electrical and electronic waste products, a treatment fund system, a qualification licensing system, and an information reporting system for treatment enterprises. Among these, the treatment fund system is an important embodiment of the extended producer responsibility system for electrical and electronic products in China. Stricter control methods and the use of environmental techniques have contributed to the notable decline in possible pollution and health risks associated with heavy metals and persistent halogenated chemicals since 2012 [66,67].

China has officially banned the import of 24 types of “foreign garbage”, including e-waste, from 1 January 2018, which has stirred a global response. In particular, since then no e-waste has been able to be legally imported to China in the name of metal scraps via formal imports [11]. Regulated enterprises have to adopt the best available recycling technologies, including manual dismantling with safety precautions and mechanical treatment. This has led to a widespread shift towards formal e-waste dismantling methods as opposed to informal methods like primitive acid leaching and open burning. For example, physical–mechanical procedures have been widely used in processing obsolete printed circuit boards [68].

The “Implementation Plan for Promoting Green Consumption” that was released in January 2022 by the National Development and Reform Commission together with seven other agencies emphasized the active implementation of the “Internet + recycling” concept [69]. By incorporating the idea, tools, and methods of internet into the recycling process, this plan aimed to promote the recycling of waste household appliances, consumer electronics, and other consumer durables [70]. Internet recycling platforms offer online and offline services, providing numerous benefits over traditional recycling methods. By promoting information disclosure, internet recycling can effectively reduce the knowledge gap/imbalance between customers and sellers. Additionally, residents can schedule door-to-door recycling more efficiently and conveniently using online platforms. Ultimately, internet recycling is more standardized, ensuring that recycled resources follow authorized disposal routes and that recyclers provide expert services to the residents [65].

As the second largest economy globally, China plays a crucial role in the transition/shift to a low-carbon and green economy. In an effort to combat climate change, China has set targets to reach peak carbon emission by 2030 and achieve carbon neutrality by 2060. Strengthening the effectiveness of the official e-waste recycling process is essential to meeting these goals [71]. Implementing the goals of peak carbon emission and carbon neutrality can be facilitated by the actions of the e-waste recycling sector. Compared to developed nations, China faces challenges in transitioning to carbon neutrality earlier, given its lower energy utilization efficiency and greater reliance on the energy derived from fossil fuels in its energy consumption structure. Economic vulnerabilities are also a concern, as China’s economic development level is lower than that of advanced economics like the US and the EU. Additionally, China lacks advanced low- and zero-carbon technologies, necessitating a three-stage process to achieve deep decarbonization: carbon peak, carbon slowdown, and carbon neutrality (or “three stages, four steps”) [72]. In the realm of industrial ecology, eco-industrial parks (EIPs) are gaining recognition as a standardized approach to support circular economy, advocate for green growth, and enhance resource efficiency [73]. It could be used predictably to improve the e-waste system in both developing and developed nations. While EIPs are not yet extensively used in the e-waste recycling sector, particularly in many developing nations, it has been demonstrated that there are two prevalent EIPs in China. In summary, China still needs significant effort in achieving its carbon emission targets. While China has established a legal framework for managing e-waste, there are still challenges that need to be thoroughly addressed to implement appropriate solutions.

## 8. Conclusions

E-waste is one of the waste streams that grows the fastest all over the world, which has caused constant public concern and conversation. China has enacted significant regulations and guidelines to manage this issue consistently. In any case, due to the deficient implementation of pertinent guidelines, obsolete treatment innovation, an absence of productive gathering framework and a low degree of public cooperation, China is still in its outset with regards to powerful and proficient e-waste management. It is foreseeable that there will be more kinds and quantities of electronic items available for our convenience, and the corresponding e-waste assets will likewise show a rising trend. In that capacity, China’s e-waste management system ought to answer and match with this trend by coordinating monetary, legal aspects, consumer patterns, and public awareness.

## Figures and Tables

**Table 1:** Environmental impact of e-waste in China.

Environmental Media	Environmental Impact
Soil	Heavy metals can be absorbed by plants through their roots and then transferred to the stem and leaf.
	Soils from e-waste dismantling are known to release PCBs, PAHs, and PCDD/Fs into the environment.
Air	Severe PM and POPs pollution in China’s e-waste recycling zones since the 2010s.
Water	Pb concentration in the water from Guiyu reached 0.4 mg/L, which exceeded 8 times the limit.
	The concentrations of metals in the groundwater were significantly higher in the e-waste areas compared to non-e-waste areas in south China.
	In the Lianjiang river, running through Guiyu, PFAS were detected, which was found to be significantly more contaminated in the water in the Guiyu e-waste recycling area than in Haojiang.
	PBDEs, PBB, and PBT were detected in all the water and sediment samples in Taizhou, indicating ubiquitous contamination in the aquatic environment in Taizhou.
Positive aspect	Recycling e-waste reuses resources, which can reduce carbon dioxide emissions and mitigate climate warming.
	More jobs were created.

**Table 2:** China’s key legislations on e-waste in recent decades.

Year	Name of Regulation	Significance of Regulation
1990	Basel Convention	Control of transboundary movements of hazardous wastes and their disposals
1995	Law on the Prevention of Environmental Pollution by Solid Wastes	Recycling and disposal of toxic and hazardous solid wastes, including e-waste, are regulated
2000	Notification on Issues Associated with the Import of the Seventh Category of Waste	Most e-waste is banned from entering China
2003	Measures for the Administration of Pollution Prevention and Control of Electronic Information Products	Restrictions on toxic and hazardous substances contained in electronic products
2005–2007	Regulations on the Administration of the Recycling and Disposal of Waste Electrical and Electronic Equipment	Promote the recycling of renewable resources and regulate the development of recycling industry
2007	Management Measures for Environmental Pollution Prevention and Control of Electronic Waste	Regulate the dismantling and disposal of e-waste
2008	Regulations on the Management of Recycling and Disposal of Waste Electrical and Electronic Products	Regulate the recycling and treatment activities of electrical and electronic waste products, promote comprehensive utilization of resources and the development of circular economy, protect the environment and human health
2009	Circular Economy Promotion Law of People’s Republic of China	EPR (Extended Producer Responsibility) was issued
2009–2011	Home Appliance Old for New Rebate Program	Old appliances go straight to formal e-waste collectors
2011	E-waste Recycling and Disposal Directive	Formal recycling activities are more strongly regulated
2013	Comprehensive Remediation Scheme of E-waste Pollution in Guiyu Town of Shantou City	Improved illegal e-waste economy in Guiyu
2017	Implementation Plan for Prohibiting the Entry of Foreign Garbage and Promoting the Reform of the Solid Waste Import Management System	Completely cut off overseas e-wastes into China
2020	Peak carbon emission by 2030 and carbon neutrality by 2060	Advocate low-carbon life to cope with climate change
2022	Implementation Plan for Promoting Green Consumption	Proposed active implementation of “Internet + recycling” concept

## Data Availability

The data presented in this study are available on request from the corresponding authors.

## Supplementary Materials

The following supporting information can be downloaded at: https://www.mdpi.com/article/10.3390/toxics12120867/s1, Figure S1: Total amount and growth rate of e-waste categorized by product type; Figure S2: Flow/distribution patterns of global e-waste.
