# Supplementary material for: Challenges of E-Waste Dismantling in China
Source: Toxics. 2024 Nov 28;12(12):867. doi: 10.3390/toxics12120867 (PMC11679424; doi:10.3390/toxics12120867)
Supplement: Supplementary file 1 [file toxics-12-00867-s001.zip › toxics-3315957-supplementary.pdf]

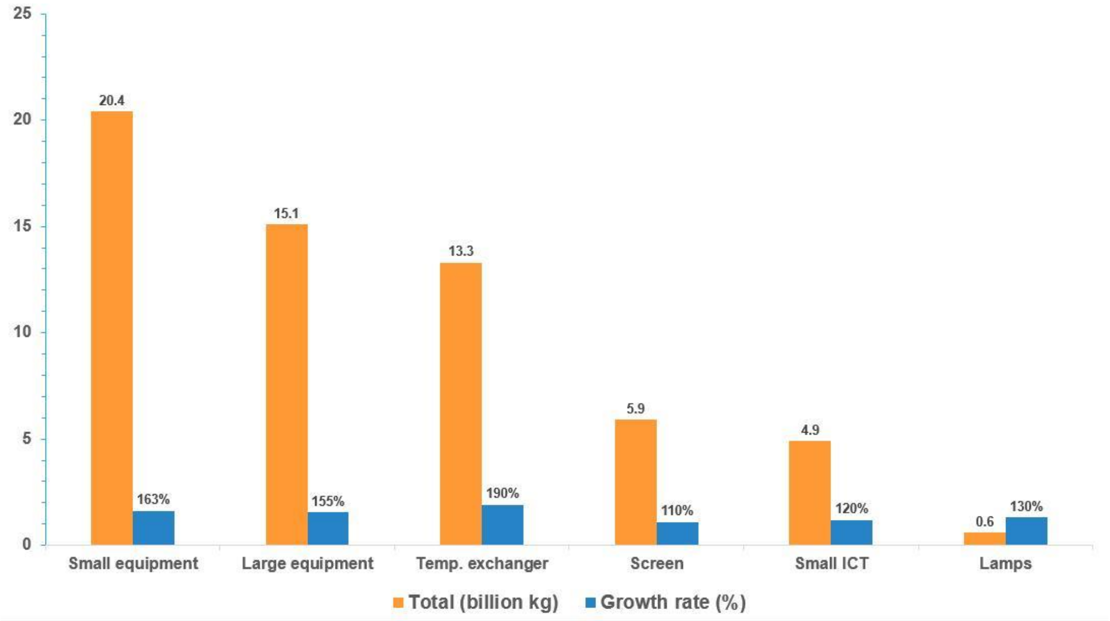

Figure S1. Total amount and growth rate of e-waste categorized by product type. Small equipment includes household items such as microwave, toaster, vacuum cleaner, calculator, electric kettle, electrical toy, and small medical device; Large equipment includes electric stove, washing machine, Xerox machine, and photovoltaic panel; Temperature exchanger includes refrigerator, air conditioner and heat pump; Screen includes computer, laptop, television, notebook and tablet; Small ICT includes mobile phone, landline telephone, router, table printer, global positioning system, and personal computer; Lamp include fluorescent, high intensity discharge and LED lamp.

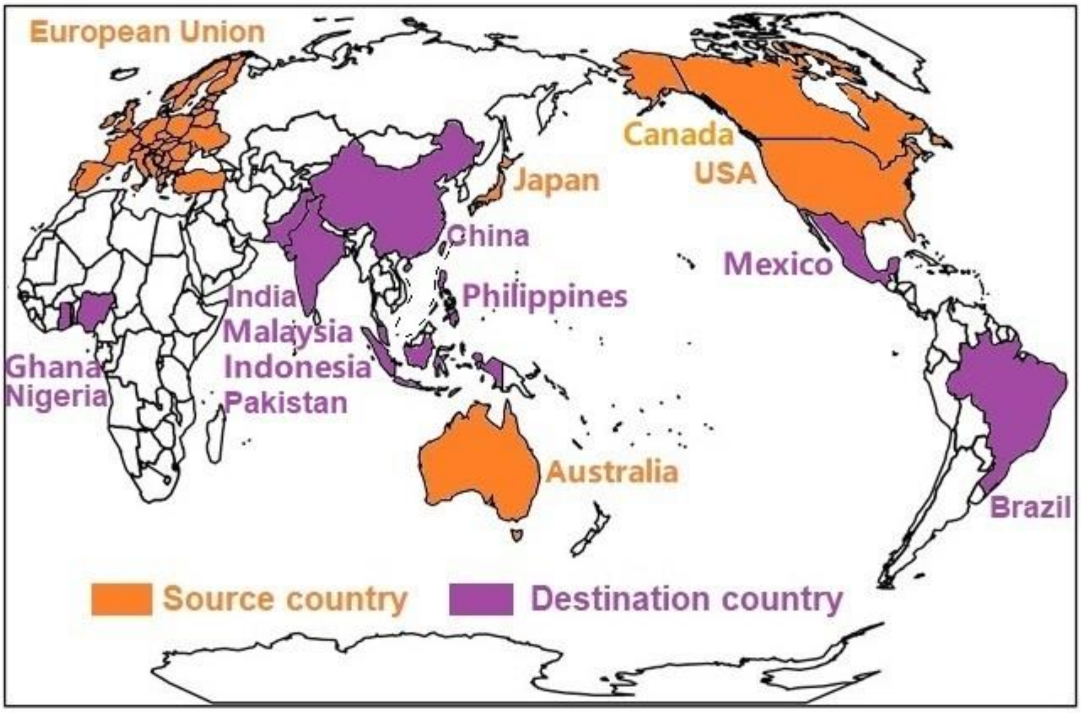

Figure S2. Flow/distribution patterns of global e-waste (Main source countries: USA, Canada, Australia, and European Union; Main destination countries: China, India, Malaysia, Indonesia, Philippines, Ghana, Nigeria, and Mexico).
